# Unipolar Barrier Near‐Infrared 2D Photodetectors for 3D Image Sensors

**DOI:** 10.1002/advs.77017

**Published:** 2026-08-10

**Authors:** Jung Pyo Hong, Sang‐Jun Kim, Jisu Jang, Je‐Jun Lee, Seong‐Jun Han, Eungseon Yeon, Jung Woo Kim, Jongtae Ahn, Sang Yeon Kim, Hyun Woo Ko, Sunghyun Han, Seung Ah Lee, Paul Hongsuck Seo, Young‐Woo Suh, Kimoon Lee, Gunuk Wang, Dong‐Guang Zheng, Sunghee Hong, Sangin Hahn, Seon Kyu Yoon, Min‐Chul Park, Do Kyung Hwang

**Affiliations:** ^1^ Center for Quantum Technology Post‐Silicon Semiconductor Institute Korea Institute of Science and Technology (KIST) Seoul Republic of Korea; ^2^ KU‐KIST Graduate School of Converging Science and Technology Korea University Seoul Republic of Korea; ^3^ Department of Electrical and Electronic Engineering Yonsei University Seoul Republic of Korea; ^4^ Department of Electric Engineering Display & Semiconductor Engineering Pukyong National University Busan Republic of Korea; ^5^ Department of Physics Changwon National University Changwon Republic of Korea; ^6^ Department of Computer Science and Engineering Korea University Seoul Republic of Korea; ^7^ Department of Mechanical Engineering Seoul National University Seoul Republic of Korea; ^8^ Department of Ophthalmology Korea University College of Medicine Guro Hospital Seoul Republic of Korea; ^9^ Department of Physics Kunsan National University Gunsan Republic of Korea; ^10^ Department of Electronic and Communication Information Engineering College Hangzhou Dianzi University Hangzhou China; ^11^ Hologram Research Center Korea Electronics Technology Institute (KETI) Seoul Republic of Korea; ^12^ Spatial Optical Information Research Center Korea Photonics Technology Institute (KOPTI) Gwangju Republic of Korea; ^13^ Division of Nanoscience & Technology, KIST School University of Science and Technology (UST) Seoul Republic of Korea

**Keywords:** 2D materials, 3D imaging, photodetectors, unipolar barriers

## Abstract

Photodetectors sensitive to the near‐infrared (NIR) range are of great importance for applications in machine vision, autonomous driving, and three‐dimensional (3D) imaging under low‐light or adverse weather conditions. Recently, two‐dimensional (2D) semiconductors have emerged as promising light‐absorbing materials for NIR detection. However, the inevitable majority‐carrier injection from the electrode fundamentally leads to high dark current and poor light‐detection capability, which has constrained the development of 2D semiconductor photodetectors so far. Here, we demonstrate a high‐detectivity NIR photodetector based on a MoTe_2_/WSe_2_/Cl–SnSe_2_ unipolar barrier heterostructure. The WSe_2_ layer acts as an efficient majority‐carrier‐blocking barrier, effectively suppressing dark current and achieving a room‐temperature specific detectivity of 6.75 × 10^8^ Jones, a linear dynamic range of 62 dB, and an effective −3 dB bandwidth approaching 0.1 MHz under 1310 nm illumination. Using this device as an imager, we show 3D NIR imaging of real objects under low‐illumination conditions, highlighting its potential for applications such as vein visualization and material identification.

## Graphical abstract

A MoTe_2_/WSe_2_/Cl–SnSe_2_ unipolar barrier photodetector is demonstrated, in which the WSe_2_ layer effectively blocks majority‐carrier injection, yielding suppressed dark current and enhanced near‐infrared (NIR) sensing. The device achieves high detectivity, a wide linear dynamic range, and fast response under 1310 nm illumination, enabling high‐fidelity 3D NIR imaging under low‐light conditions.

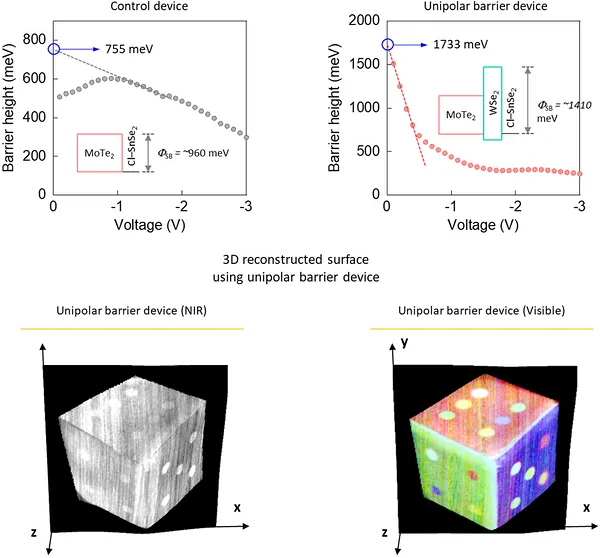

## 1 Introduction

Near‐infrared (NIR) photodetectors based on two‐dimensional (2D) semiconductors have attracted considerable attention owing to their potential in diverse emerging applications, such as optical communication, advanced imaging, and light detection and ranging (LiDAR) technologies [1, 2, 3, 4, 5, 6]. One of the key performance metrics for NIR photodetectors is the specific detectivity (*D* ^*^) [7], which quantifies the ability of a detector to sense weak optical signals over noise, and can be expressed as:

(1) $$D^{*}=\frac{R\sqrt{A}}{i_{n}}\;$$

where *R* is the responsivity, *A* is the active area of the photodetector, and *i*_n_ is the noise current spectral density with a unit of A Hz^−1/2^. This expression clearly highlights the importance of minimizing the noise current spectral density to achieve high detectivity in the photodetectors. In general, the dominant noise sources depend on the operating regime: under photovoltaic mode (under zero bias), thermal noise is usually the main limitation, whereas in photoconductive mode (under reverse bias), a reduction in dark current suppresses shot noise, which in turn enhances *D* ^*^ [7, 8].

In practice, however, most NIR 2D photodetectors employing narrow‐bandgap absorbers (e.g., MoTe_2_) usually suffer from large reverse‐bias dark current, primarily due to majority‐carrier injection across small energy barriers (Figure 1a). This dark‐current pathway increases the noise current spectral density and severely degrades the *D* ^*^. To address this challenge, unipolar barrier structures have been introduced as a promising strategy (Figure 1b) [8, 9, 10, 11, 12]. These structures selectively block majority carriers through large band offsets in either the conduction or valence band, while maintaining a nearly barrier‐free transport pathway for photogenerated carriers in the opposite band. As a result, the dark current can be effectively suppressed without significantly compromising photocarrier extraction, leading to enhanced detectivity (Figure 1c).

**FIGURE 1:**
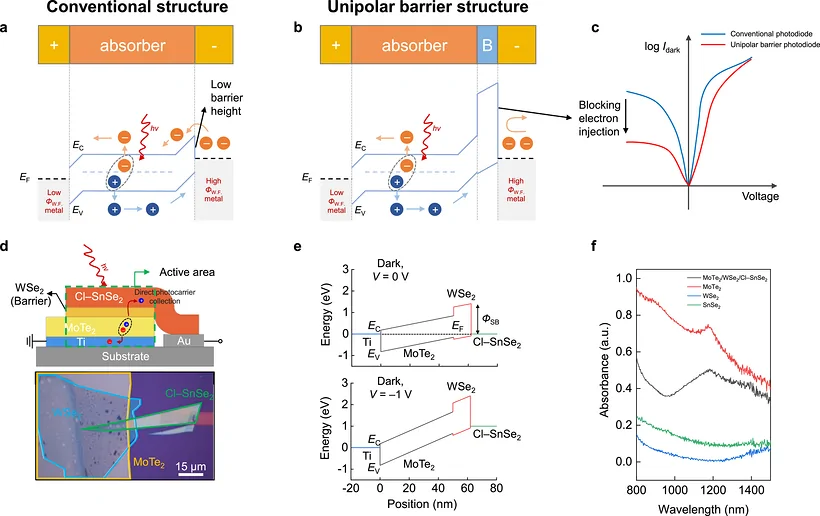
Design of the unipolar barrier NIR photodetector. (a, b) Schematic energy‐band diagrams of conventional (a) and unipolar barrier (b) NIR photodetectors under reverse bias. The unipolar barrier blocks injected majority electrons, while photocarriers can flow unimpeded. *E*_F_, *E*_V_, and *E*_C_ correspond to the Fermi level, valence band maximum, and conduction band minimum, respectively. (c) Schematic *I*–*V* curves of diodes with (red) and without (blue) a unipolar barrier. (d) Schematic illustration and optical microscope image of unipolar barrier MoTe_2_/WSe_2_/Cl–SnSe_2_ device. In the schematic, the green dashed region denotes the active heterojunction area. The corresponding active area was estimated to be 113 µm^2^ from the optical microscope image. (e) Simulated band diagrams of MoTe_2_/WSe_2_/Cl–SnSe_2_ heterostructure at *V* = 0 and −1 V. (f) Absorption spectra of MoTe_2_, WSe_2_, Cl–SnSe_2_, and their heterostructure.

The unipolar barrier concept has been widely explored in infrared photodetector architectures, where selective carrier blocking is used to reduce dark current and noise while preserving photocurrent transport [9, 10, 12]. In these systems, the absorber/barrier/contact band alignment must be carefully engineered so that the barrier blocks majority‐carrier injection without impeding the collection of photogenerated carriers. However, in conventional semiconductor platforms, the absorber, barrier, and contact layers are often selected from a limited range of materials that can form high‐quality interfaces with suitable band offsets. This constraint makes it difficult to independently tune the carrier‐blocking barrier and the photocarrier transport pathway.

2D van der Waals heterostructures provide a particularly attractive platform for implementing unipolar barrier photodetectors because they offer dangling‐bond‐free surfaces, tunable band structures, and relaxed lattice‐matching constraints. A pioneering study demonstrated 2D unipolar barrier photodetectors based on band‐engineered van der Waals heterostructures, including WS_2_/h–BN/PdSe_2_ nBn and black phosphorus/MoS_2_/graphene pBp architectures, thereby extending the unipolar barrier concept to 2D material systems [8]. Subsequent work further advanced this direction by demonstrating a graphene/WSe_2_/PtSe_2_ unipolar barrier heterostructure with high‐performance photodetection characteristics [13]. These studies established 2D unipolar barrier engineering as an effective strategy for suppressing dark current and improving photodetector sensitivity.

Despite these advances, previous 2D unipolar barrier photodetectors have mainly focused on demonstrating carrier‐selective band engineering and high‐performance photodetection. However, how the barrier design affects reverse‐bias dark‐current suppression, the effective transport barrier height, and detectivity based on measured noise spectral density has not been fully clarified in vertical 2D unipolar barrier photodetectors. Therefore, it is important to develop and evaluate a vertical 2D unipolar barrier platform that suppresses reverse‐bias dark current while maintaining efficient NIR photocarrier extraction and low‐noise signal readout.

Herein, we report a high‐detectivity NIR 2D unipolar barrier photodetector based on a vertically stacked MoTe_2_/WSe_2_/Cl–SnSe_2_ heterostructure. In this device, MoTe_2_ serves as the NIR absorber, WSe_2_ functions as an electron‐blocking unipolar barrier, and Cl–SnSe_2_ acts as a 2D contact layer for vertical carrier collection. The WSe_2_ barrier introduces a large electron barrier that suppresses majority‐carrier injection and reverse‐bias dark current, while maintaining an efficient hole‐transport pathway for photocarrier extraction. By measuring the noise spectral density, we demonstrate that the unipolar barrier design improves the *D* ^*^ from 1.69 × 10^7^ to 6.75 × 10^8^ Jones under 1310 nm laser illumination. Moreover, the improved NIR photodetection performance enables proof‐of‐concept 3D NIR imaging, highlighting the potential of 2D unipolar barrier photodetectors for future optoelectronic and sensing platforms.

## 2 Results and Discussion

### 2.1 Design of the Unipolar Barrier Photodetectors

As depicted in Figure 1d, unipolar barrier MoTe_2_/WSe_2_/Cl–SnSe_2_ photodetectors were fabricated by mechanical exfoliation and a dry transfer method (See Figure S1 for atomic force microscopy (AFM) measurements). The absorber layer, MoTe_2_ (∼51 nm), which exhibits strong light absorption in the NIR range [14, 15, 16], was transferred onto a pre‐patterned low‐work‐function Ti electrode, followed by vertical stacking of a WSe_2_ barrier layer (∼12 nm) and high‐work‐function 2D metal Cl–SnSe_2_ (∼30 nm). Additionally, Cl–SnSe_2_ was contacted with an Au electrode, which allows Fermi‐level pinning‐free contact [17, 18, 19, 20] and convenient probing during the device measurements. All 2D layers were sequentially stacked on the bottom Ti electrode to form a fully vertical heterostructure, enabling efficient out‐of‐plane carrier collection and minimizing parasitic resistance by eliminating the quasi‐neutral region [2, 15]. In the device schematic, the active heterojunction area is outlined by the green dashed region. The corresponding active area was estimated to be ∼113 µm^2^ from the optical microscope image. Furthermore, SCAPS simulations were performed to verify that the designed energy‐band configuration indeed exhibits a unipolar barrier (details are provided in Table S1). The simulated band diagrams of this heterostructure are presented in Figure 1e. At *V* = 0 V, the theoretical electron barrier height from the Cl–SnSe_2_ to WSe_2_ barrier layer is 1410 meV, whereas the hole barrier is 91 meV, approaching nearly zero, and this band design is consistent with Figure 1b, indicating that this band structure inhibits electrons from the Cl–SnSe_2_ electrode, whereas photocarriers generated from the MoTe_2_ absorber can contribute to the photocurrent. This clearly illustrates a well‐aligned unipolar electron barrier in the MoTe_2_/WSe_2_/Cl–SnSe_2_ heterostructure under both zero‐ and reverse‐bias conditions. To further demonstrate the NIR absorption capability of the aforementioned heterostructure, we conducted absorption spectra measurements of the corresponding heterostructure (See Figure S2 for an optical image of the heterostructure). As shown in Figure 1f, this heterostructure and MoTe_2_ exhibit strong NIR absorption (gray and red lines, respectively), while WSe_2_ and Cl–SnSe_2_ (blue and green lines, respectively) show relatively weak absorption, highlighting the suitability of this heterostructure for NIR photodetection applications.

### 2.2 Characterizations of the Unipolar Barrier Photodetectors

Using unipolar barrier architecture, we fabricated NIR photodetectors and investigated the fundamental characteristics of MoTe_2_/WSe_2_/Cl–SnSe_2_ unipolar barrier devices. First, we measured the dark current‐voltage (*I*–*V*) characteristics of control MoTe_2_/Cl–SnSe_2_ and unipolar barrier MoTe_2_/WSe_2_/Cl–SnSe_2_ photodetectors, as presented in Figure 2a. At *V* = −1.0 V, the dark‐saturation current of the unipolar barrier photodetector is approximately two orders of magnitude lower than that of the control device (a statistical analysis of saturation currents for both device types is shown in Figure S3), which is attributed to the presence of the unipolar electron barrier. Under reverse bias, the dark current can originate from multiple transport mechanisms, including majority‐carrier injection, surface leakage current, generation–recombination (G–R) current, field‐assisted tunneling currents, and intrinsic diffusion current. In a properly aligned electron‐blocking unipolar barrier, dark‐current components requiring electron transport across the barrier are suppressed, whereas photogenerated holes can flow through the nearly barrier‐free valence‐band pathway [8, 9, 12, 21]. In our MoTe_2_/WSe_2_/Cl–SnSe_2_ heterostructure, the simulated band alignment in Figure 1e indicates that the WSe_2_ layer forms a large electron barrier while maintaining a small hole barrier, providing a physical basis for the reduced reverse‐saturation current.

**FIGURE 2:**
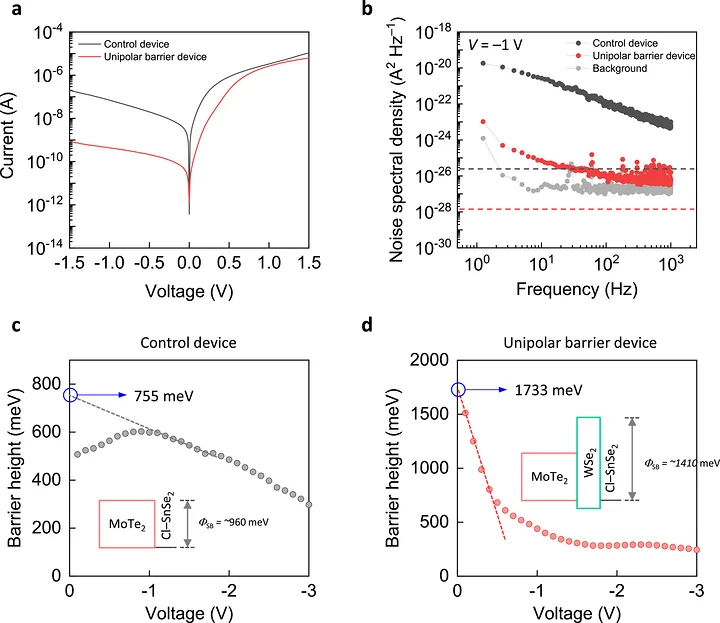
Characterization of unipolar barrier photodetector. (a) Dark *I*–*V* characteristics of control (black) and unipolar barrier MoTe_2_/WSe_2_/Cl–SnSe_2_ (red) photodetectors. (b) Current‐noise power spectral density of control MoTe_2_/Cl–SnSe_2_ and unipolar barrier MoTe_2_/WSe_2_/Cl–SnSe_2_ photodetectors measured at *V* = −1 V. The gray line with markers represents the background noise floor of the measurement system. The black and red dashed lines indicate the shot‐noise limits of the control and unipolar barrier photodetectors, respectively. (c, d) Extracted barrier heights of the control MoTe_2_/Cl–SnSe_2_ (c) and unipolar barrier MoTe_2_/WSe_2_/Cl–SnSe_2_ (d) photodetectors as a function of reverse‐bias voltage. The effective barrier heights were obtained from the *y*‐intercepts of linear fits (black and red dashed lines). Insets: corresponding energy‐band scheme for each device type.

As mentioned above, since separating the signal from noise is critical for photodetection, minimizing the noise current is essential [7]. To further investigate, we measured the noise spectral density of both unipolar barrier and control devices under no illumination at *V* = −1 V. As shown in Figure 2b, the current‐noise power spectral density (*S*_n_) of the unipolar barrier MoTe_2_/WSe_2_/Cl–SnSe_2_ photodetector decreases with increasing frequency owing to the lower‐frequency (1/*f*) noise component and approaches the background noise floor of the measurement setup at approximately 1 kHz. At this frequency, *S*_n_ is 1.5 × 10^−26^ A^2^ Hz^−1^, corresponding to a noise current spectral density of $i_{n}=\sqrt{S_{n}}=$ 1.22 × 10^−13^ A Hz^−1/2^. The dashed black and red lines denote the shot‐noise limits of the control and unipolar barrier device, respectively. These results indicate that the unipolar barrier suppresses leakage current under reverse bias, thereby reducing the noise level and contributing to enhanced detectivity. We attribute this suppression of reverse‐saturation currents to carefully engineered energy‐band alignment, in which injected majority carriers are effectively blocked by the unipolar electron barrier. The reproducibility of the low‐noise characteristics was further confirmed by measuring the current‐noise power spectral densities of five independently fabricated unipolar barrier devices (Figure S4). To further verify the effectiveness of the unipolar barrier, we intentionally fabricated a misaligned unipolar hole barrier MoTe_2_/MoS_2_/Cl–SnSe_2_ diode, which blocks both injected and photogenerated hole carriers (See Figure S5a for the energy‐band diagram scheme). As shown in Figure S5b, this device exhibits a slightly lower reverse‐saturation current compared with the control MoTe_2_/Cl–SnSe_2_ photodetector, owing to the hole‐blocking unipolar barrier under dark conditions. However, its optoelectronic performance, including the open‐circuit voltage and overall photocurrent (such as short‐circuit current) is severely degraded under the same illumination condition, as presented in Figure S5c, confirming that the hole barrier hinders photocarrier collection. These results further validate that our unipolar electron barrier design can suppress reverse‐dark current while allowing photocarriers to flow unimpeded. Furthermore, temperature‐dependent *I*–*V* measurements were performed to estimate the effective barrier height of the control and unipolar barrier devices (See Figure S6 for plots) [22, 23]. Figures 2c,d show the effective barrier height as a function of voltage for each device. The effective barrier heights extrapolated to *V* = 0 V are 755 meV for the control device and 1733 meV for the unipolar barrier device, compared with the SCAPS‐simulated values of 960 and 1410 meV, respectively, as shown in Figure 1e. The quantitative differences arise because the SCAPS simulation provides an idealized one‐dimensional band profile based on nominal material parameters and uniform interfaces, whereas the experimentally extracted values represent effective transport barriers in the actual devices. The experimentally extracted effective barriers can therefore be influenced by real‐device factors that are not fully captured in the idealized simulation, including variations in material properties and unintentional doping, interface electrostatics at van der Waals contacts, local barrier inhomogeneity, and thermally activated contact or interface transport [20, 22, 24]. These factors can shift the apparent activation barrier in either direction. In the control device, lower‐energy leakage pathways and interface inhomogeneity can reduce the apparent barrier height relative to the simulated value. In the unipolar barrier device, the insertion of the WSe_2_ layer introduces an additional electron‐blocking transport bottleneck, and interfacial electrostatic effects can increase the experimentally extracted effective barrier relative to the idealized band offset. The finite temperature range used for the Arrhenius analysis and the extrapolation to *V* = 0 V may also contribute to the quantitative difference. Nevertheless, both the experimental and simulated results consistently show a substantial increase in the electron barrier height after insertion of the WSe_2_ layer, supporting the electron‐blocking band alignment predicted by the SCAPS simulation. Together with the two‐order‐of‐magnitude reduction in reverse‐saturation current shown in Figure 2a, these results support the effective suppression of injected majority electrons by the WSe_2_ layer. Previous studies have shown that properly aligned unipolar barriers can additionally suppress surface‐leakage and reinjected‐current components by blocking carrier pathways that differ from that of the photocurrent. Junction‐related G–R and tunneling currents may also be mitigated when depletion‐region formation is minimized [8, 9, 12, 21]. In our device, these effects may provide additional contributions to the reduced dark current. Based on the simulated band alignment, the increased effective barrier height, and the comparison with the intentionally misaligned hole‐barrier device, the blocking of injected majority electrons by the WSe_2_ barrier is identified as the primary experimentally supported mechanism underlying the observed dark‐current suppression.

### 2.3 Characterization of Photodetector

We systematically characterized the electrical and optoelectronic performance of both control and unipolar barrier photodetectors under visible and NIR light illumination. Under 532 nm (visible) and 1310 nm (NIR) laser illumination, the unipolar MoTe_2_/WSe_2_/Cl–SnSe_2_ devices exhibited superior photodetection characteristics, including a higher linear dynamic range (LDR), enhanced photoresponsivity, and increased *D* ^*^, compared to the control MoTe_2_/Cl–SnSe_2_ photodetectors under identical conditions. This improvement originates from the suppressed reverse‐dark‐saturation current in the unipolar barrier structure, which reduces the noise current and enables clear signal separation from noise at low‐light levels.

As shown in Figure 3a and Figure S7a, under 532 nm illumination, the unipolar barrier photodetectors exhibited an extended linear photoresponse range from 141.4 µW to 4.7 W cm^−2^, whereas the control devices operated only within a narrow range. This confirms that reduced reverse‐saturation current allows reliable detection at lower illumination levels. A similar trend was observed under 1310 nm illumination (Figure 3d and Figure S8a), where the unipolar barrier devices maintained a broad detection range from 871.4 µW to 16.3 W cm^−2^. Figure 3b and Figure S7b plot the photocurrent at *V* = −1 V as a function of incident power, showing significantly improved linearity for the unipolar barrier devices (72 dB) compared with the control devices (42 dB). Likewise, under 1310 nm illumination, the LDR was enhanced from 37 dB (control) to 62 dB (unipolar barrier), as shown in Figure 3e and Figure S8b. The LDR was calculated according to:

(2) $$\mathrm{LDR}\;=20\log\left(\frac{I_{high}}{I_{low}}\right)$$

where *I*_high_ and *I*_low_ are the highest and lowest photocurrent within the linear response regime. To further evaluate the effect of the unipolar barrier on photosensitivity, we compared the photoswitching ratios of the control and unipolar barrier devices under reverse bias (Figure S9). The photoswitching ratio was defined as |*I*_light_|/|*I*_dark_|, where *I*_light_ and *I*_dark_ are the currents measured under illumination and dark conditions, respectively. At *V* = −1 V under 1310 nm illumination, the photoswitching ratios of the control and unipolar barrier devices were approximately 1.14 and 8.00, respectively, at a power density of ∼100 mW cm^−2^ (Figure S9a). At a higher power density of ∼20 W cm^−2^, the corresponding values increased to approximately 3.4 × 10^1^ and 2.9 × 10^3^, respectively (Figure S9b). These results indicate that suppression of the reverse dark current substantially improves light‐to‐dark signal discrimination in the unipolar barrier device. Figure 3c and Figure S7c further summarize the responsivity and *D* ^*^ as a function of light intensity for both device types. The unipolar barrier devices exhibited enhanced responsivity, which can be attributed to suppressed noise current and improved carrier collection efficiency [12]. Furthermore, the linear responsivity across a broad range of light intensities provides additional evidence of efficient carrier extraction [17]. Since practical photodetectors are typically operated under weak illumination rather than strong laser power, responsivity alone is insufficient to evaluate device performance; the noise must also be considered, making D^*^ a reliable figure of merit.

**FIGURE 3:**
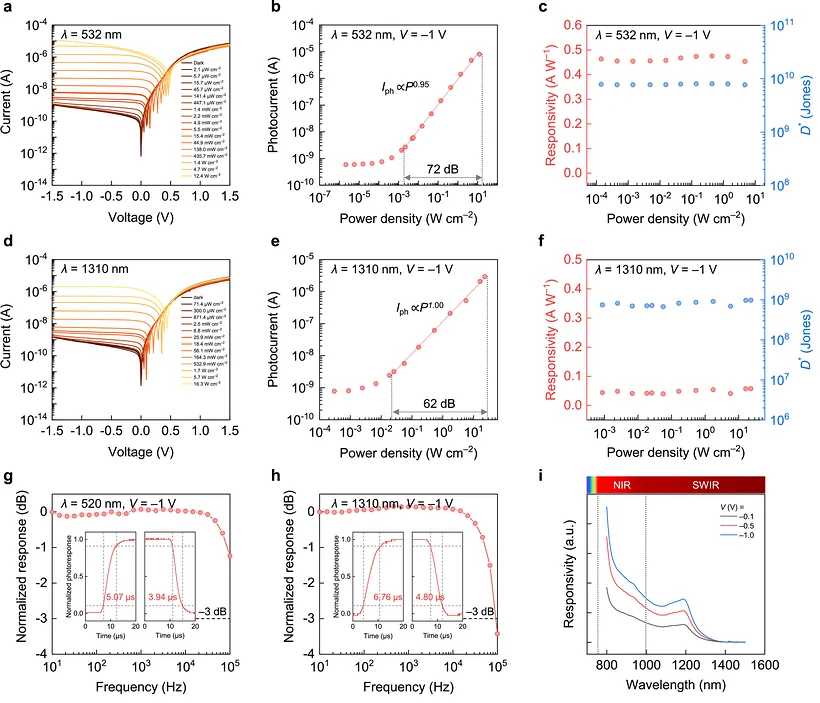
Characterization of the unipolar barrier photodetectors under visible and NIR illumination. (a, d) *I*–*V* curves of the unipolar barrier photodetectors measured under 532 (a) and 1310 nm (d) laser illumination with varying power densities. (b, e) Photocurrent as a function of light power density at *V* = −1 V under 532 (b) and 1310 nm (e) illumination. The estimated LDR of the device is 72 and 62 dB, respectively. The red lines represent power‐law fits to experimental data. (c, f) Responsivity and *D* ^*^ as a function of light power density under the corresponding illumination conditions. (g, h) Normalized photoresponse as a function of modulation frequency under 520 and 1310 nm laser illumination. In both cases, the −3 dB cutoff frequency approaches 100 kHz. (i) Spectral responsivity of the photodetector measured at three different reverse‐bias voltages.

We extracted D^*^ as a function of incident power (Figure 3c and Figure 3f), confirming that the unipolar barrier devices maintain high‐responsivity under both visible and NIR illumination at *V* = −1 V. Specifically, *D* ^*^ increased from 6.04 × 10^8^ to 8.03 × 10^9^ Jones under 532 nm excitation and from 1.69 × 10^7^ to 6.75 × 10^8^ Jones under 1310 nm laser illumination at ∼100 mW cm^−2^. These results clearly demonstrate the improved sensitivity of the unipolar barrier devices to weak optical signals, consistent with the photocurrent and LDR data shown in Figure 3b,e. For the *D* ^*^ calculation, the noise current spectral density of *i*_n_ = 1.22 × 10^−13^ A Hz^−1/2^, extracted at 1 kHz from Figure 2b, was used. The frequency of 1 kHz was selected as the onset of the background‐noise‐limited regime. It should be noted that ambiguously defined active areas can lead to over‐ or underestimation of responsivity and detectivity. Thus, careful definition of the active area is essential. In our design, the WSe_2_ barrier layer may also contribute to the photocurrent under 532 nm illumination. To determine the effective area, we performed photocurrent mapping (Figure S10), confirming that the vertically stacked heterojunction region predominantly contributes to the photocurrent.

Additional thickness‐dependent measurements were performed using thin (∼5 nm) and thick (>30 nm) WSe_2_ barrier devices to clarify the role of WSe_2_ thickness in photodetector performance (Figure S11). The thin WSe_2_ device showed incomplete reverse‐bias dark current suppression, as supported by the band schematic, optical image, and 1310 nm power‐dependent *I*–*V* characteristics in Figure S11a,c,e. In contrast, the thick WSe_2_ device effectively suppressed the dark current but exhibited a pronounced bias‐dependent response under 1310 nm illumination, indicating reduced photocarrier collection caused by inefficient hole extraction (Figure S11b,d,f). These results indicate that the ∼12 nm WSe_2_ barrier used in the main device provides a favorable trade‐off between suppressing reverse‐bias dark current and maintaining efficient NIR photoresponse.

Frequency photoresponse measurements (Figure 3g,h) revealed that the unipolar barrier photodetectors operate at high speed, with a −3 dB bandwidth approaching 100 kHz under both visible and NIR illumination; these values are comparable to the corresponding −3 dB bandwidths measured from the control devices (Figures S7d and S8d). The optical frequency‐response measurement setup is shown in Figure S12. To further examine whether this frequency response originates from the device rather than solely from the measurement setup, additional capacitance‐frequency (*C*–*F*) measurements were performed at *V* = −1 V in the dark. The *C*–*F* characteristics showed a clear capacitance roll‐off in the range of approximately 50–100 kHz (Figure S13), which is consistent with the optical −3 dB frequency and the µs‐scale transient response. These results indicate that the measured bandwidth reflects the effective device response, including the RC‐related electrical response of the vertical heterostructure. The corresponding transient photocurrent responses (insets of Figure 3g,h) show rise and fall times on the order of a few microseconds (5.07 and 3.94 µs for rise and fall for visible illumination, and 6.76 and 4.80 µs under NIR illumination), further supporting the µs‐scale response dynamics. Notably, the control devices exhibited nearly identical response speeds, indicating that the inserted unipolar barrier layer does not hinder the device response speed, while still providing substantial improvements in responsivity and detectivity. To evaluate the spectral detection range of the unipolar MoTe_2_/WSe_2_/Cl–SnSe_2_ photodetector, we measured the spectral responsivity under different reverse‐bias voltages (Figure 3i). The spectral responsivity varied with reverse bias and extended up to approximately 1400 nm. Notably, the maximum responsivity was observed near 800 nm, consistent with the absorbance spectrum of the MoTe_2_/WSe_2_/Cl–SnSe_2_ heterostructure shown in Figure 1f. To further verify photoresponse near this responsivity maximum region, we additionally measured the power‐dependent photoresponse under 808 nm laser illumination (Figure S14). The photocurrent increased linearly with incident light power at *V* = −1 V, with LDR of 66 dB, confirming efficient photocarrier generation and extraction. Taken together, these results underscore the strong NIR photoresponse of the unipolar barrier photodetector. To further benchmark the device performance, we compared the MoTe_2_/WSe_2_/Cl–SnSe_2_ photodetector with representative unipolar barrier photodetectors based on 2D heterostructures (Table S2). The comparison shows that the present device exhibits a competitive combination of low dark current, measured‐noise‐based detectivity, broad LDR, and µs‐scale response, particularly under 1310 nm illumination.

### 2.4 Near‐Infrared 3D Imaging Demonstration

To evaluate the NIR characteristics of the unipolar barrier MoTe_2_/WSe_2_/Cl–SnSe_2_ photodetectors with high signal‐to‐noise ratio (SNR) and *D* ^*^, 2D imaging was performed and subsequently extended to 3D visualization. In this context, photometric stereo was employed as the visualization approach [25]. Figure 4a illustrates the principle of photometric stereo using a commercial image sensor, along with example images captured under varying illumination directions. The acquired images showed direction‐dependent brightness variations. These brightness variations were used to estimate surface normals, which were then integrated to generate the 3D visualization [26, 27, 28].

**FIGURE 4:**
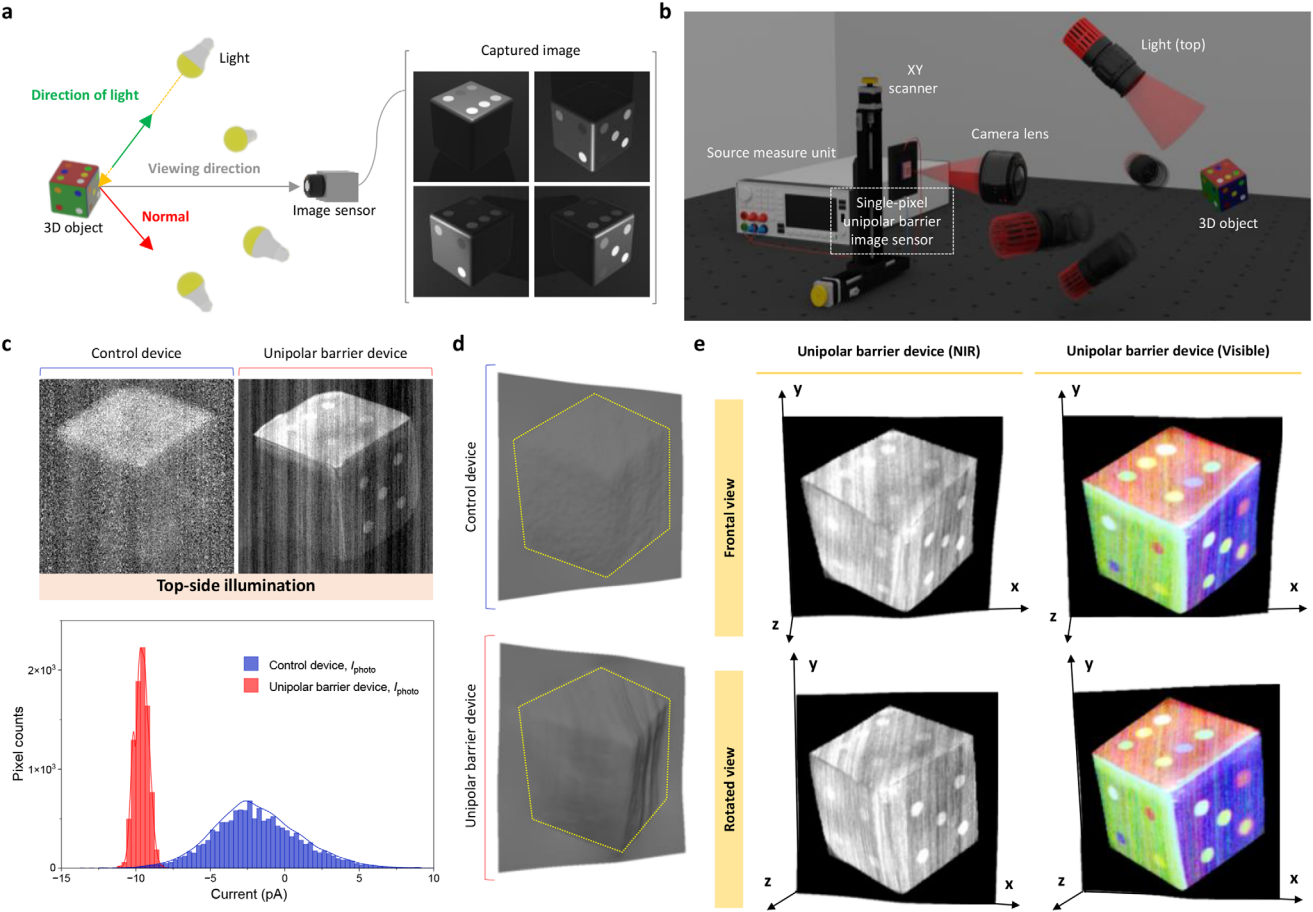
NIR 3D imaging using unipolar barrier MoTe_2_/WSe_2_/Cl–SnSe_2_ photodetectors. (a) Principle of photometric stereo image acquisition. (b) Schematic of the 3D imaging system using unipolar barrier MoTe_2_/WSe_2_/Cl–SnSe_2_ photodetectors as single‐pixel image sensors. (c) Images (top) and histograms of photocurrent distributions (bottom) of the control MoTe_2_/Cl–SnSe_2_ and unipolar barrier MoTe_2_/WSe_2_/Cl–SnSe_2_ photodetectors under NIR illumination. The 2D images have a spatial resolution of 220 × 220 pixels. (d) 3D surface reconstructions of control and unipolar barrier photodetectors. The dashed outline indicates the region of interest for analysis. (e) Final 3D renderings with textures mapped onto the reconstructed surfaces under NIR (left) and visible (right) illumination. The renderings demonstrate the depth perception and surface continuity of the 3D object.

The 3D imaging system illustrated in Figure 4b was implemented based on the photometric stereo method, and a unipolar barrier single‐pixel photodetector was used as the image sensor. The object, camera lens, and image sensor were fixed and aligned along the same optical axis to maintain imaging stability [29]. Four NIR light sources (850 nm) arranged around the lens were sequentially activated, effectively revealing the reflective properties of the object surface under different illumination conditions [30, 31]. The reflected light was focused onto the image sensor through the lens. The image sensor was mounted on an XY motorized stage, allowing scanning to acquire surface information. In particular, signal stability was enhanced by averaging data sampled three times at each pixel position, and contrast enhancement was applied to obtain NIR images with improved visibility [32]. A photograph of the experimental setup is shown in Figure S15, and further information on the experiments is provided in the Experimental Methods.

Figure 4c presents 2D images and photocurrent distributions acquired under top‐side NIR illumination using control MoTe_2_/Cl–SnSe_2_ and unipolar barrier MoTe_2_/WSe_2_/Cl–SnSe_2_ photodetectors. In the 2D imaging results, the control device, which has no unipolar barrier, exhibited a weak photoresponse, making it difficult to distinguish the contours of the object. This limitation arose from the low detectivity, where photocurrents overlapped with noise currents under low‐light intensity, thereby preventing reliable signal discrimination. In contrast, the unipolar barrier photodetectors, owing to low dark current and enhanced detectivity, exhibited clearly separated signal distributions, resulting in a distinct brightness contrast under the same illumination conditions. Consequently, the contours and surface structures of the object were clearly reproduced, reflecting improved signal discrimination and structural fidelity relative to the control photodetectors. The 2D imaging results obtained under other illumination directions are provided in Figure S16, and the corresponding acquisition parameters are summarized in Table S3. For each illumination direction, a 220 × 220 pixel image was acquired using a single‐pixel scanning configuration. Because the present system requires sequential spatial X–Y scanning, the demonstrated imaging approach is intended as a proof‐of‐concept for validating the device‐level low‐light NIR sensing capability rather than as a real‐time imaging platform. The photocurrent histograms represent the statistical distribution of pixel‐level photocurrents measured from the illuminated surface during imaging. Unlike the control device, the unipolar barrier device showed a narrower distribution and higher photocurrent. This result indicates that noise suppression led to improved pixel uniformity, enabling stable photocurrent measurements even under weak illumination and confirming the potential of the proposed photodetectors as device‐level sensing elements for low‐light applications.

The difference in photocurrent characteristics directly affected the 3D reconstruction results. Figure 4d shows the photometric stereo reconstruction, where the control device produced a dice image with indistinct boundaries and an irregular surface, whereas the unipolar barrier device yielded distinct contours and an accurate representation of fine details (Figure S17). Furthermore, based on the reconstructed vertex height, the control device reproduced about 30% of the object height, whereas the unipolar barrier device achieved a reconstruction fidelity of approximately 79% (Figure S18). To achieve realistic visualization, texture information was further applied to the reconstructed shapes (Figure S19) [33]. As shown in Figure 4e, the unipolar barrier photodetectors enabled successful texture mapping of reconstructed surfaces under both NIR and visible illumination. In each result, the top image corresponds to the frontal view, while the bottom image shows the same scene from a rotated viewpoint, confirming the spatial depth and continuity of the reconstructed surfaces. The proposed unipolar barrier MoTe_2_/WSe_2_/Cl–SnSe_2_ photodetectors demonstrated brightness detection under low‐light conditions through enhanced SNR. This capability improved the accuracy of photometric stereo reconstruction, leading to enhanced 3D shape quality. Furthermore, the photodetectors exhibited stable photoresponse not only under NIR illumination but also under visible‐light conditions (Figure S20), supporting their broad spectral responsivity. Although the present single‐pixel scanning configuration is limited to stationary‐object imaging because of its long acquisition time, these results establish a device‐level basis for future low‐light sensing and next‐generation optoelectronic imaging platforms. Further development toward practical imaging systems will require detector‐array integration and parallelized readout schemes to substantially reduce the acquisition time.

## 3 Conclusion

In summary, we present a high‐performance NIR photodetector based on a MoTe_2_/WSe_2_/Cl–SnSe_2_ unipolar barrier heterostructure. The insertion of the WSe_2_ layer forms an efficient unipolar barrier that suppresses majority‐carrier injection from the contacts, reducing the dark current by two orders of magnitude. The resulting device exhibits a room‐temperature *D* ^*^ of 6.75 × 10^8^ Jones, a linear dynamic range of 62 dB, and an effective −3 dB bandwidth approaching 0.1 MHz under 1310 nm illumination. Owing to the high performance of the unipolar barrier photodetector, we further demonstrated 3D NIR imaging capable of resolving real objects even under low‐light conditions. These findings establish a viable route towards compact, low‐power, and high‐efficiency imaging and sensing technologies based on 2D materials.

## 4 Experimental Methods

### 4.1 Device Fabrication and Measurements

Both unipolar barrier MoTe_2_/WSe_2_/Cl–SnSe_2_ devices and control MoTe_2_/Cl–SnSe_2_ devices were fabricated by mechanical exfoliation and dry transfer using poly(dimethylsiloxane) (PDMS) films. A MoTe_2_ flake (∼50 nm) was transferred onto the pre‐patterned Ti electrode. For the unipolar barrier structure, a WSe_2_ layer (∼12 nm) was subsequently stacked on MoTe_2_, followed by a Cl–SnSe_2_ layer (∼30 nm), forming a fully vertical heterostructure. Cl–SnSe_2_ was also contacted with a pre‐patterned Au electrode, serving as a probing pad. All photocurrent measurements were performed under ambient conditions using an Agilent 4156B parameter analyzer. For imaging applications, a source meter (Keithley 2636B) was employed, and measurements were taken under dark and laser (Thorlabs) illuminated conditions. The optical intensity was adjusted with neutral density filters placed in the beam path and calibrated using commercial Si and Ge photodetectors. Transient photoresponse and photoelectrical bandwidth were measured with a high‐speed oscilloscope (Tektronix TDS2024) driven by a function generator (Tektronix AFG31000). Capacitance–frequency measurements were performed using an HP 4284A precision LCR meter under dark conditions at *V* = –1 V. Noise spectral density was obtained using a spectrum analyzer (ADVANTEST R9211C) coupled to a low‐noise preamplifier (Stanford Research Systems SR570) [34].

### 4.2 Synthesis of Cl–SnSe_2_

Cl–SnSe_2_ (SnSe_1.98_Cl_0.02_) was synthesized via chemical vapor transport (CVT), following a previously reported procedure [35]. In brief, powders of Sn, Se, and SnCl_2_ were mixed in stoichiometric proportions and sealed in an evacuated quartz ampoule. The reaction, ((2 – *x*)Sn + *x*SnCl_2_ + 2(2 – *x*)Se → 2SnSe_2‐_ *_x_* Cl*_x_*) was carried out in two steps. The mixture was first heated to 250°C for 24 h and then annealed at 400°C for 48 h. Subsequently, the product was melted at 750°C for 24 h and slowly cooled to room temperature to obtain high‐quality Cl–doped SnSe_2_ crystals.

### 4.3 Drift‐Diffusion Simulations

Simulations of the unipolar barrier devices were conducted using SCAPS‐1D software [36]. SCAPS is an open‐source software and can be obtained from https://users.elis.ugent.be/ELISgroups/solar/projects/scaps. The simulation parameters used in this study are presented in Table S1.

### 4.4 Imaging System Setup

The 3D imaging system consisted of an XY motorized stage (PI VT‐800), a camera lens, LED illumination, and a source measure unit (Keithley 2636B). For visible imaging, a 3000 K white LED (Thorlabs MWWHLP1) illuminated the object, and the reflected light was separated into the red, green, and blue bands using 650, 550, and 450 nm optical bandpass filters mounted in front of the sensor. For NIR imaging, an IR LED (Thorlabs M850LP1) was employed, and no additional bandpass filter was required due to its sufficiently narrow spectral bandwidth. Both visible and NIR illuminations were collimated using adapters (Thorlabs SM2F32‐A for visible and SM2F32‐B for NIR) to provide uniform illumination direction across pixels [37]. The reflected light was focused by the camera lens onto the photodetector, which was connected to the source measure unit to record the photocurrent. The photocurrent was measured at *V* = −1 V. For each illumination direction, the scan was performed over a 220 × 220‐pixel grid with a step size of 0.015 mm. The acquisition time for a single image was 66,243 s (≈18.4 h), corresponding to an effective acquisition time of approximately 1.369 s per pixel. At each pixel position, the photocurrent was measured three times and averaged. Based on the scan pitch and effective acquisition time per pixel, the effective scan rate was estimated to be approximately 10.96 µm s^−1^. This effective rate includes not only stage movement but also source‐meter‐based current measurements, instrument communication, and data storage processes.

## Author Contributions

J.P.H., S.‐J.K. and J.J. contributed equally to this work. S.K.Y., M.‐C.P., and D.K.H. supervised the project. All the authors discussed the results and approved the manuscript .

## Funding

Korea Institute of Science and Technology (KIST) Institution Program (Grant Nos. 26E0002 and 26V0500‐26‐P019), KU‐KIST School project, and the National Research Foundation of Korea (NRF) (Grant Nos. RS‐2024‐00461204 and RS‐2026‐25478068) Global Joint Research Program funded by the Pukyong National University (grant no. 202505820001), Technology Innovation Program (02316245) by the Ministry of Trade, Industry & Energy (MOTIE, Korea), the National Research Foundation of Korea (NRF) (Grant No. 2020R1F1A1070009).

## Conflicts of Interest

The authors declare no conflicts of interest.

## Supporting information




**Supporting File**: advs77017‐sup‐0001‐SuppMat.docx.

## Data Availability

The data that support the findings of this study are available from the corresponding author upon reasonable request.
