# Supplementary material for: Unipolar Barrier Near‐Infrared 2D Photodetectors for 3D Image Sensors
Source: Adv Sci (Weinh). 2026 Aug 10;13(61):e77017. doi: 10.1002/advs.77017 (PMC13455832; doi:10.1002/advs.77017)
Supplement: Supplementary file 1 [file ADVS-13-e77017-s001.docx]

Supporting Information

**Unipolar Barrier Near-Infrared 2D Photodetectors for 3D Image Sensors**

*Jung Pyo Hong*, *Sang-Jun Kim*, *Jisu Jang*, *Je-Jun Lee*, *Seong-Jun Han*, *Eungseon Yeon*, *Jung Woo Kim*, *Jongtae Ahn*, *Sang Yeon Kim*, *Hyun Woo Ko*, *Sunghyun Han*, *Seung Ah Lee*, *Paul Hongsuck Seo*, *Young-Woo Suh*, *Kimoon Lee*, *Gunuk Wang*, *Dong-Guang Zheng*, *Sunghee Hong, Sangin Hahn,* *Seon Kyu Yoon**, *Min-Chul Park**, and *Do Kyung Hwang**

**Figure S1.** AFM measurement of the unipolar barrier MoTe_2_/WSe_2_/Cl–SnSe_2_ heterostructure. a) AFM height image of MoTe_2_/WSe_2_/Cl–SnSe_2_ heterostructure in Figure 1d of the main text. b–d) Corresponding height profiles of the MoTe_2_ (b), WSe_2_ (c), and Cl–SnSe_2_ (d) layers measured along the red, blue, and green lines in (a). The thickness of each layer is approximately 51, 12, and 30 nm respectively.

**Figure S2.** Optical image of the MoTe_2_/WSe_2_/Cl–SnSe_2_ heterostructure. Optical microscope image of the MoTe_2_/WSe_2_/Cl–SnSe_2_ heterostructure corresponding to the region shown in Figure 1f. Each measurement was performed over the squared areas marked in the image.

**Figure S3.** Statistical distribution of dark-current for the control and unipolar barrier devices. The distributions are represented as box- and whisker-plots. Black and red boxes indicate the control and unipolar barrier devices, respectively. The central line within each box denotes the median, the box limits correspond to the standard deviation, and the whiskers extend to 1.5 outliers. At least ten devices were measured for each case.

**Figure S4**. Device-to-device reproducibility of the noise characteristics of the unipolar barrier photodetectors. Current-noise power spectral densities of five independently fabricated MoTe_2_/WSe_2_/Cl–SnSe_2_ unipolar barrier photodetectors measured under dark conditions at *V* = –1 V. The measurements were performed under the same experimental conditions used for the representative device shown in Figure 2b. All five devices exhibited similar noise spectra, which decrease with increasing frequency owing to the 1/*f* noise component and approach the background noise floor of the measurement setup at approximately 1 kHz.

**Figure S5.** Characterization of the misaligned unipolar barrier MoTe_2_/MoS_2_/Cl–SnSe_2_ photodetectors. a) Schematic energy band diagram of misaligned unipolar barrier photodetector under reverse-bias. b,c) *I*–*V* characteristics of control MoTe_2_/Cl–SnSe_2_ (black) and misaligned unipolar barrier MoTe_2_/MoS_2_/Cl–SnSe_2_ (blue) photodetectors measured under dark-conditions (b) and white-light illumination (c).

To investigate the influence of unipolar barrier configuration, we intentionally fabricated a misaligned unipolar barrier MoTe_2_/MoS_2_/Cl–SnSe_2_ photodetector. In contrast to the properly aligned MoTe_2_/WSe_2_/Cl–SnSe_2_ device, which blocks only injected electrons, the downward unipolar barrier formed by MoS_2_ layer blocks both injected and photoexcited hole carriers. As shown in Figure S5b, dark-saturation current of MoTe_2_/MoS_2_/Cl–SnSe_2_ device was slightly lower than the control MoTe_2_/Cl–SnSe_2_, indicating that electrons, rather than holes, dominate the dark-current in the MoTe_2_ channel. Furthermore, under identical white-light illumination (Figure S5c), the misaligned device exhibited lower photocurrent and open-circuit voltage compared with the control device, because the photoexcited holes were blocked by the downward unipolar barrier, leading to inefficient photocarrier collection. These results further confirm that the unipolar barrier design in the MoTe_2_/WSe_2_/Cl–SnSe_2_ structure is well aligned and effectively suppresses only the undesired electron injection.

**Figure S6.** Temperature-dependent *I*–*V* characteristics. a,b) Temperature-dependent *I*–*V* measurements of the control (a) and unipolar barrier (b) devices, performed from 330 to 370 K in 10 K intervals.

**Figure S7.** Visible range characterization of the control MoTe_2_/Cl–SnSe_2_ photodetectors. a) *I*–*V* curves of control MoTe_2_/Cl–SnSe_2_ photodetector measured under 532 nm laser illumination with varying power densities. b) Photocurrent of the corresponding device as a function of light power density measured at *V* = –1 V. The red line represents a power-law fit to the experimental data. The estimated linear dynamic range (LDR) of the device is 42 dB, which is lower than that of unipolar barrier MoTe_2_/WSe_2_/Cl–SnSe_2_ photodetector. c) Responsivity and *D*^*^ as a function of light power density under the same measurement conditions. d) Normalized photoresponse as a function of modulation frequency under 520 nm laser illumination.

**Figure S8.** NIR range characterization of the control MoTe_2_/Cl–SnSe_2_ photodetectors. a) *I*–*V* curves of the control MoTe_2_/Cl–SnSe_2_ photodetector measured under 1310 nm laser illumination with varying power densities. b) Photocurrent of the corresponding device as a function of light power density measured at *V* = –1 V. The red line represents a power-law fit to the experimental data. The estimated LDR of the device is 37 dB, which is lower than that of unipolar barrier MoTe_2_/WSe_2_/Cl–SnSe_2_ photodetectors. c) Responsivity and *D*^*^ as a function of light power density under corresponding condition. d) Normalized photoresponse as a function of modulation frequency under 1310 nm laser illumination.

**Figure S9.** Comparison of the photoswitching ratios of the control and unipolar barrier photodetectors. Photoswitching ratios of representative control MoTe_2_/Cl–SnSe_2_ and unipolar barrier MoTe_2_/WSe_2_/Cl–SnSe_2_ photodetectors measured at *V* = –1 V under 1310 nm illumination with power densities of ~100 mW cm^–2^ (a) and 20 W cm^–2^ (b). The higher photoswitching ratios of unipolar barrier device indicate the dark-current suppression improves light-to-dark signal discrimination.

**Figure S10.** Photocurrent mapping of the unipolar barrier MoTe_2_/WSe_2_/Cl–SnSe_2_ device. a) Optical microscope image of MoTe_2_/WSe_2_/Cl–SnSe_2_ unipolar barrier device. b,c) Photocurrent mapping results under 532 (b) and 904 (c) nm laser illumination. The spot size of laser is 3 μm.

**Figure S11.** Thickness-dependent characteristics of MoTe_2_/WSe_2_/Cl–SnSe_2_ unipolar barrier photodetectors. a,b) Schematic band diagrams of the devices with thin (a) and thick (b) WSe_2_ barrier layers, respectively. c,d) Optical microscope images of the corresponding thin (c) and thick (d) WSe_2_ devices, respectively. e,f) 1310 nm power-dependent *I*–*V* characteristics of the thin (e) and thick (f) WSe_2_ devices, respectively.

To examine the effect of WSe_2_ barrier thickness on device performance, MoTe_2_/WSe_2_/Cl–SnSe_2_ photodetectors with thin (~5 nm) and thick (>30 nm) WSe_2_ barrier layers were additionally fabricated and characterized. The thin WSe_2_ device exhibited a reverse-bias dark current lower than that of the control MoTe_2_/Cl–SnSe_2_ device but higher than that of the optimized ~12 nm WSe_2_ device, indicating that the thin WSe₂ layer partially suppresses majority-carrier injection but does not fully block leakage current. This behavior is attributed to incomplete electron blocking and possible tunneling-assisted leakage through the thin barrier. In addition, further reducing the WSe_2_ thickness toward the mono- or bilayer regime may alter the intended unipolar band alignment because the WSe_2_ band gap increases with decreasing layer thickness, potentially disturbing the desired combination of a large electron barrier and a nearly barrier-free hole-transport pathway. In contrast, the thick WSe_2_ device maintained a low dark current but showed a strongly bias-dependent response under 1310 nm illumination. This behavior is attributed to inefficient hole extraction because the increased WSe_2_ thickness lengthens the hole-transport pathway, while the finite valence-band offset at the MoTe_2_/WSe_2_ interface can act as a local hole-transport bottleneck. This comparison supports the selection of the ~12 nm WSe_2_ barrier as a balanced thickness for suppressing dark current while maintaining an efficient NIR photoresponse.

**Figure S12.** Schematic of the bandwidth and response time measurement system in the main text.

**Figure S13.** Capacitance-frequency characteristics of unipolar barrier MoTe_2_/WSe_2_/Cl–SnSe_2_ device. Frequency-dependent capacitance measured under dark conditions. The capacitance remains nearly constant at low frequencies and exhibits a clear roll-off in the range of approximately 50–100 kHz, as indicated by the shaded region.

**Figure S14.** Photoresponse characteristics of the MoTe_2_/WSe_2_/Cl–SnSe_2_ unipolar barrier photodetector under 808 nm laser illumination. a) Power-dependent *I*–*V* characteristics measured under 808 nm laser illumination. b) Photocurrent as a function of incident light power density at *V* = –1 V. The red line represents a power-law fit to the experimental data. The LDR of the device is 66 dB.

**Figure S15.** Photograph of the experimental setup for 3D imaging.

**Figure S16.** 2D imaging results under different NIR illumination directions. a) Images acquired using the unipolar barrier MoTe_2_/WSe_2_/Cl–SnSe_2_ photodetector. b) Images acquired using the control MoTe_2_/Cl–SnSe_2_ photodetector.

2D imaging results obtained under different NIR illumination directions are shown for the unipolar barrier and control devices. The images were acquired through a scanning process using a single-pixel photodetector. The scanning was performed over a 220 × 220 pixels grid with a step size of 0.015 mm, and the total imaging time was 66,243 s (≈18.4 h). The effective acquisition time per pixel and effective scan rate were estimated to be approximately 1.369 s per pixel and 10.96 μm s^–1^, respectively. The key imaging acquisition parameters are summarized in Table S3.

**Figure S17.** Gaussian filtering applied to reconstructed 3D surfaces. a) Original 3D reconstruction result using unipolar barrier MoTe_2_/WSe_2_/Cl–SnSe_2_ photodetector. b) Gaussian-filtered 3D result.

The figure shows the effect of Gaussian surface smoothing on the photometric stereo 3D reconstructions. Figure S17a presents the original reconstruction result, in which the overall object shape and height profile are represented. Figure S17b presents the Gaussian-filtered result. The smoothing operation effectively suppressed surface irregularities and enhanced the continuity of the reconstructed geometry, enabling clearer visualization of the overall object structure.

**Figure S18.** Comparison of reconstructed 3D models with the reference dice geometry.

The schematic compares the 3D reconstruction results obtained from the unipolar barrier and control devices, focusing on the apex vertex formed at the intersection of three adjacent faces of the dice. For geometric comparison, three base vertices opposite to the apex vertex of each reconstructed model were aligned with the corresponding vertices of the dice, ensuring consistent spatial registration. A reference plane was then defined by these three aligned vertices, and the relative height of the remaining apex vertex was measured with respect to this plane. The red and blue curves represent the reconstructed boundary contours obtained from the unipolar barrier and control devices. In this configuration, the vertices corresponding to the dice, unipolar barrier, and control reconstructions were labeled A, B, and C. The vertical distances between these vertices were measured to compare their relative heights. The relative vertex heights were determined to be 7.18 mm for A, 5.63 mm for B, and 2.13 mm for C. These results indicate that the unipolar barrier device reconstructed the dice geometry with improved structural accuracy and height fidelity compared with the control device. All alignment, height measurement, and visualization processes were conducted and rendered in Blender based on the reconstructed 3D data.

**Figure S19.** Texture Image Acquisition. a) Texture image under NIR illumination. b) Texture image under visible illumination**.**

The texture map for realistic surface visualization was generated using four images captured under varying illumination directions in the photometric stereo experiment. Each image exhibited partial shading and brightness nonuniformities depending on the illumination conditions. To obtain a uniformly illuminated texture, a pixel-wise maximum intensity composite method was applied. The maximum pixel intensity among the images was selected for each pixel to produce a single texture map, which was implemented in Python. This process compensated for shadows and occluded regions, yielding a uniform and visually consistent texture representation of the object surface. Under visible illumination, due to the relatively bright lighting and the tilt of the dice, no additional processing was required, and the image captured under top-side illumination was used as the texture map.

**Figure S20.** Acquisition of full-color 3D images.

The experimental setup and image acquisition procedure were the same as those described for the NIR 3D imaging measurements. Under visible-light conditions, four light sources positioned around the camera lens (top, bottom, left, and right) illuminated the object. The reflected light was spectrally separated into red, green, and blue components using optical bandpass filters and subsequently recombined to generate full-color images. The reconstructed images had a spatial resolution of 220 × 220 pixels. The color contrast and reflective characteristics of the object varied depending on the illumination direction. The unipolar barrier image sensor successfully captured not only the contours of the dice but also the color variations of multicolored pips.

**Table S1.** Multilayer MoTe_2_ and WSe_2_ parameters for SCAPS-1D simulation

| **Materials** | **MoTe_2_** | | **WSe_2_** | |
| --- | --- | --- | --- | --- |
| **Parameter** | **Value** | **Ref.** | **Value** | **Ref.** |
| Thickness (nm) | 50 |  | 12 |  |
| Bandgap (eV) | 1.0 | 1 | 1.5 | 3 |
| Electron affinity (eV) | 4.02 | 1 | 3.61 | 3 |
| Dielectric permittivity | 13 | 2 | 13.8 | 4 |
| Density of states in conduction band, *N*_C_ (cm^–3^) | 3 × 10^18^ | 2 | 8.3 × 10^18^ | 4 |
| Density of states in valence band, *N*_V_ (cm^–3^) | 4 × 10^18^ | 2 | 1.6 × 10^19^ | 4 |
| Out-of-plane mobility (for electrons /holes) (cm^2^ V^–1^ s^–1^) | 110/426 | 2 | 100/500 | 4 |
| Donor concentration, *N*_D_ (cm^–3^) | 1 × 10^17^ | 2 |  |  |
| Acceptor concentration, *N*_A_ (cm^–3^) |  |  | 1× 10^16^ | 4 |

| **Device Structure** | **Dark current** | **Dark current rectification ratio** | **Detectivity (Jones)** | **Rise time** | **Fall time** | **LDR (dB)** | **Operation wavelength (nm)** | **Ref.** |
| --- | --- | --- | --- | --- | --- | --- | --- | --- |
| **MoTe_2_/WSe_2_/Cl–SnSe_2_** | **600 pA (–1 V)** | **4 × 10^3^** | **8.03 × 10^9^** | **5.07 μs** | **3.94 μs** | **72** | **532** | **This work** |
| **MoTe_2_/WSe_2_/Cl–SnSe_2_** | **600 pA (–1 V)** | **4 × 10^3^** | **6.75 × 10^8^** | **6.76 μs** | **4.80 μs** | **62** | **1310** | **This work** |
| b-AsP/WS_2_/b-AsP | 1 nA (–0.1 V) | 4 | - | - | -- | - | 4600 | 5 |
| WS_2_/hBN/PdSe_2_ | 100 pA (–0.5 V) | 1 × 10^3^ | - | 74 μs | 26 μs | 66 | 520 | 6 |
| BP/MoS_2_/Gr | 80 μA (–1 V) | 3 | - | - | - | - | Blackbody radiation | 6 |
| Bi_2_S_3_/WS_2_/Bi_2_S_3_ | 0.3 nA (–5 V) | 2 | 3 × 10^11^ | 2.66 ms | 7.48 ms | 32 | 532 | 7 |
| b-AsP/MoS_2_/BP | 2 μA (–1 V) | 6 | - | - | - | - | 1550 | 8 |
| InSe/SnS_2_/GeSe | 10 pA (–1 V) | 1 × 10^3^ | - | 7.3 μs | 8.9 μs | 26 | 532 | 9 |
| Gr/WSe_2_/PtSe_2_ | 20 nA (–1 V) | 1 × 10^3^ | 6.41 × 10^10^ | 669 ns | 452 ns | 60 | 520 | 10 |

**Table S2.** Performance comparison of representative unipolar barrier photodetectors

**Table S3.** Acquisition parameters for NIR single-pixel scanning

| **Parameter** | **Value** |
| --- | --- |
| Image size | 220 × 220 pixels |
| Total number of pixels | 48,400 |
| Step size | 0.015 mm |
| Total imaging time | 66,243 s (≈ 18.4 h) |
| Effective acquisition time per pixel | 1.368657 s pixel^–1^ |
| Effective scan rate | 10.9596 μm s^–1^ |

**References**

1. B. -S. Yu et al. “Reversible Polarity Control in 2D MoTe_2_ Field-Effect Transistors for Complementary Logic Gate Applications.” *Adv. Funct. Mater.* 34 (2024): 2404129.
2. M. Moustafa, T. AlZoubi “Effect of the n-MoTe_2_ interfacial layer in cadmium telluride solar cells using SCAPS.” *Optik* 170 (2018): 101.
3. J. Ahn et al. “Near-Infrared Self-Powered Linearly Polarized Photodetection and Digital Incoherent Holography Using WSe_2_/ReSe_2_ van der Waals Heterostructure.” *ACS Nano* 15 (2021): 17917.
4. R. Chaurasiya, G. K. Gupta, A. Dixit “Ultrathin Janus WSSe buffer layer for W(S/Se)_2_ absorber based solar cells: A hybrid, DFT and macroscopic, simulation studies.” *Sol. Energy Mater. Sol. Cells* 201 (2019): 110076.
5. W. Deng, M. Dai, C. Wang, C. You, W. Chen, S. Han, J. Han, F. Wang, M. Ye, S. Zhu “Switchable Unipolar-Barrier Van der Waals Heterostructures with Natural Anisotropy for Full Linear Polarimetry Detection.” *Adv. Mater*. 34 (2022): 2203766
6. Y. Chen et al. “Unipolar Barrier Photodetectors Based on van der Waals Heterostructures.” *Nat. Electron.* 4 (2021): 357.
7. S. Shen et al. “Unipolar Barrier Engineering in 1D/2D Hybrid Van der Waals Heterostructures for High-Performance Broadband Polarization-Sensitive Photodetection.” *Small* 9 (2026): e12087
8. S. Zhang et al. “Multi-dimensional optical information acquisition based on a misaligned unipolar barrier photodetector” *Nat. Commun*. 15 (2024): 7071
9. Y. Yan et al. “Unipolar barriers in near-broken-gap heterostructures for high-performance self-powered photodetectors” *Appl. Phys. Lett.* 122 (2023): 043505
10. S. Wang et al. “Unipolar Barrier Photodetectors Based on Van Der Waals Heterostructure with Ultra-High Light On/Off Ratio and Fast Speed” *Adv. Sci*. 12 (2025): 2413844
